# A report of two children with Gorham-Stout disease

**DOI:** 10.1186/s12887-019-1561-0

**Published:** 2019-06-24

**Authors:** Mario Edgar Tena-Sanabria, Larisa Yarindy Jesús-Mejenes, Gabriela Fuentes-Herrera, Félix Alejandro Álvarez-Martínez, Nora Patricia Victorio-García, Juan Carlos Núñez-Enríquez

**Affiliations:** 1 Unidad Médica de Alta Especialidad, Servicio de Ortopedia Pediátrica, Hospital de Pediatría “Dr. Silvestre Frenk Freund”, Centro Médico Nacional “Siglo XXI”, Instituto Mexicano del Seguro Social, Mexico City, Mexico; 2 Unidad de Investigación en Epidemiología Clínica, Hospital de Pediatría “Dr. Silvestre Frenk Freund”, Centro Médico Nacional “Siglo XXI”, Instituto Mexicano del Seguro Social, Avenida Cuauhtémoc 330. Colonia Doctores Delegación Cuauhtémoc C.P, 06720 Mexico City, Mexico

**Keywords:** Gorham-stout disease, Idiopathic osteolysis, Children, Pediatric orthopedics

## Abstract

**Background:**

Gorham-Stout disease is a rare condition characterized by unifocal and massive type IV osteolysis (variant of idiopathic nonhereditary osteolytic disease) with a slow progression, which is self-limiting for some years. It is characterized by recurrent vascular tumors with disruption of the anatomical architecture and intraosseous proliferation of vascular channels that leads to the destruction and resorption of the bone matrix. The aim of this study is to present the clinical features of this disease, as well as the importance of prompt diagnosis and treatment, with a review of the reported cases.

**Case reports:**

We describe two cases of Gorham-Stout disease between 2013 and 2017 with surgical interventions, follow-up and results. Case one involves an 11-year-old male with involvement of the left iliac bone, with adequate evolution after a surgical procedure with a lyophilized cadaveric tricortical bone allograft. Case two involves a 6-year-old male with cervical spine C1-C3 repercussion; in the protocol for surgical treatment, he presented with signs of spinal cord compression and died.

**Conclusion:**

Diagnosis of Gorham-Stout disease is made by exclusion, and its clinical presentation varies widely, from spontaneous remission to a fatal outcome.

**Electronic supplementary material:**

The online version of this article (10.1186/s12887-019-1561-0) contains supplementary material, which is available to authorized users.

## Background

Gorham-Stout disease is a rare condition characterized by unifocal and massive type IV osteolysis (a type of idiopathic nonhereditary osteolytic disease) with a slow progression, which is self-limiting in some years. It is characterized by recurrent vascular tumors with disruption of the anatomical architecture and intraosseous proliferation of vascular channels that leads to the destruction and resorption of the bone matrix [1, 2]. (see Table 1).

**Table 1:** Classification of “idiopathic osteolysis” according to Hardegger et al

Type	Typical age at manifestation	Location of the manifestation	Nephropathy	Prognosis
I Hereditary multicentric osteolysis with dominant inheritance	Juvenile	Carpotarsal osteolysis, sometimes affecting the radius and ulna	No	Good, self-limiting in adolescence
II Hereditary multicentric osteolysis with recessive transmission	Juvenile	Consistent with type I, in addition to generalized osteoporosis	No	Good, self-limiting in adolescence
III Nonhereditary multicentric osteolysis with nephropathy	Juvenile	Mainly carpometacarpal, tarsal involvement is rare, malignant hypertension	Yes, proteinuria in progressive renal pathology	Unfavorable
IV Gorham-Stout syndrome	Independent of age	Typical: shoulder, pelvis, facial skull bones	No	Usually good. When there is spinal involvement or chylothorax, mortality rises more than 50%
V Winchester syndrome (hereditary, autosomal recessive)	Juvenile	Carpotarsal osteolysis and contractures, short stature, osteoporosis, corneal deterioration	No	Progressive

Gorham-Stout disease was initially described by Jackson in 1838 and later classified by Gorham and Stout in 1955 [3, 4]. Worldwide, considering all the potential affected regions only 200 cases have been reported. Osteolysis can affect any bone. It is associated with functional deficits and pain [5]. The most frequently described anatomical locations in Gorham-Stout disease are the thorax, femur, mandible, pelvis, scapula, humerus, vertebra, tibia, clavicle, and joints [6].

The clinical course varies widely, from spontaneous remission to progressive osteolysis with some mild clinical manifestations to a clinical course with a fatal outcome. An average mortality of 13% has been described [7]. The fatal outcome is usually related to the presence of chylothorax or spinal instability caused by osteolytic destruction of the vertebrae, with a mortality range of 33 to 53% [7, 8]. The classic radiologic features of Gorham-Stout disease are tapering bone ends or mouse tail appearance. Histopathological findings include lymphatic and vascular tissue in the bone and D2–40 immunohistochemistry positivity, with a sensitivity of 92.6% and specificity of 98.8% [9].

Treatment modalities include pharmacological treatment with bisphosphonates, vitamin D, and biological drugs; surgical treatment; radiotherapy; or a combination of these options. Surgical options include bone resection, resection with endoprosthetic reconstruction, and resection with biological reconstruction (bone graft) [10–17].

Our aim was to present the clinical features of two Mexican pediatric cases with this rare condition.

## Case reports

We described two Mexican children with Gorham-Stout disease who were treated between 2013 and 2017 in the Pediatric Orthopedic Department of a tertiary referral level pediatric hospital in Mexico City.

## Case presentation no. 1

A 7-year-old male patient began his condition in 2008 with pain and claudication in the left lower limb. He was taken to his Family Medical Unit, where he was referred to the Emergency Unit of the Lomas Verdes High Specialty Medical Unit (UMAE) of Traumatology and Orthopedics of the Mexican Institute of Social Security (IMSS), where they performed a bone biopsy and curettage with bone graft application in lyophilized cadaveric tricortical bone allograft, with a presumptive diagnosis of aneurysmal bone cyst. He remained under surveillance in the private sector; however, pain persisted in the left lower limb. Afterwards, he presented with pain exacerbation, which was why he returned to the emergency department in 2013.

**Fig. 1:**
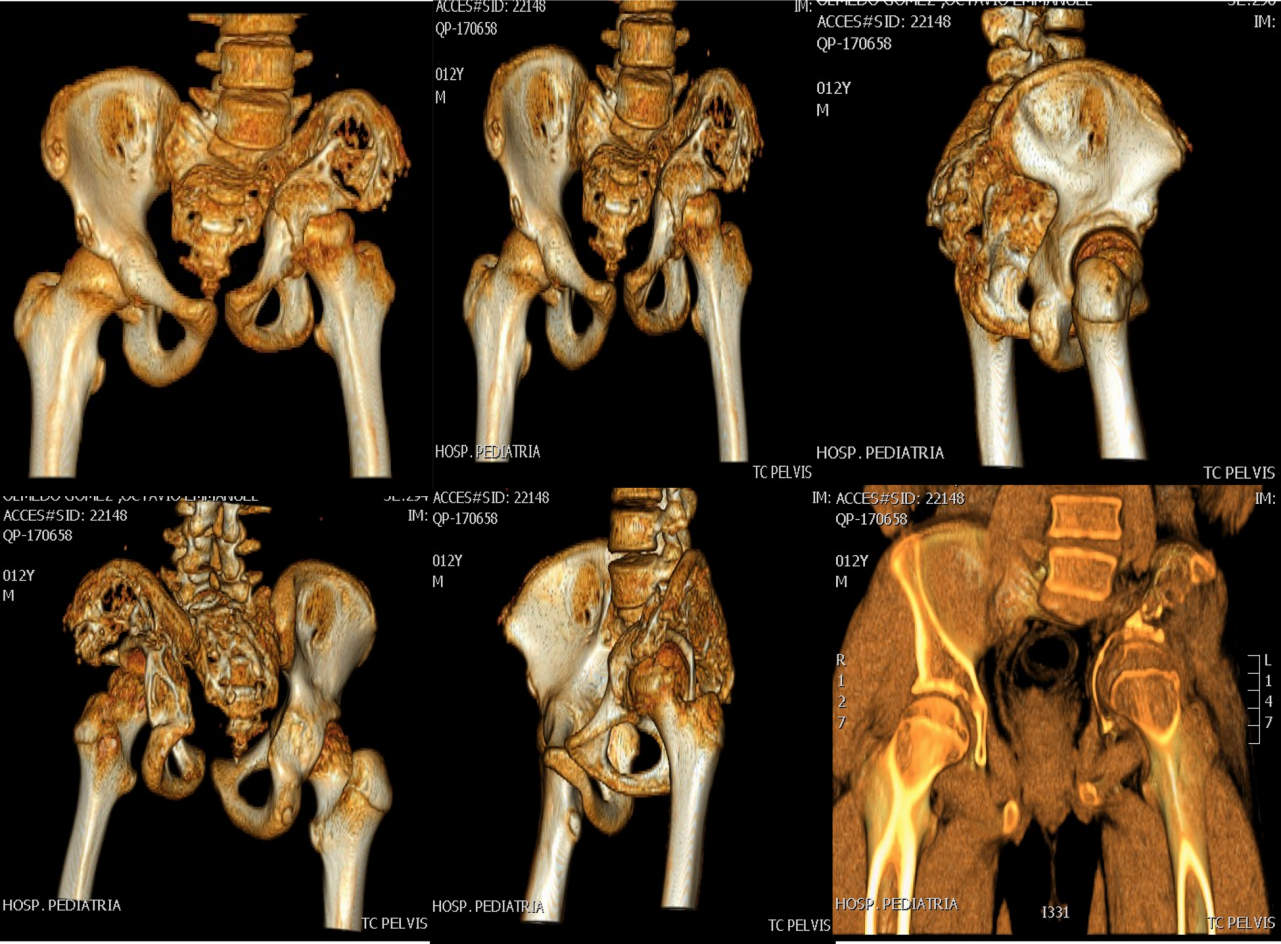
Tomographic images of the pelvis with three-dimensional reconstruction showing extensive bone destruction of the left sacroiliac joint and left iliac bone that leads to a posterosuperior displacement of the ipsilateral coxofemoral joint

**Fig. 2:**
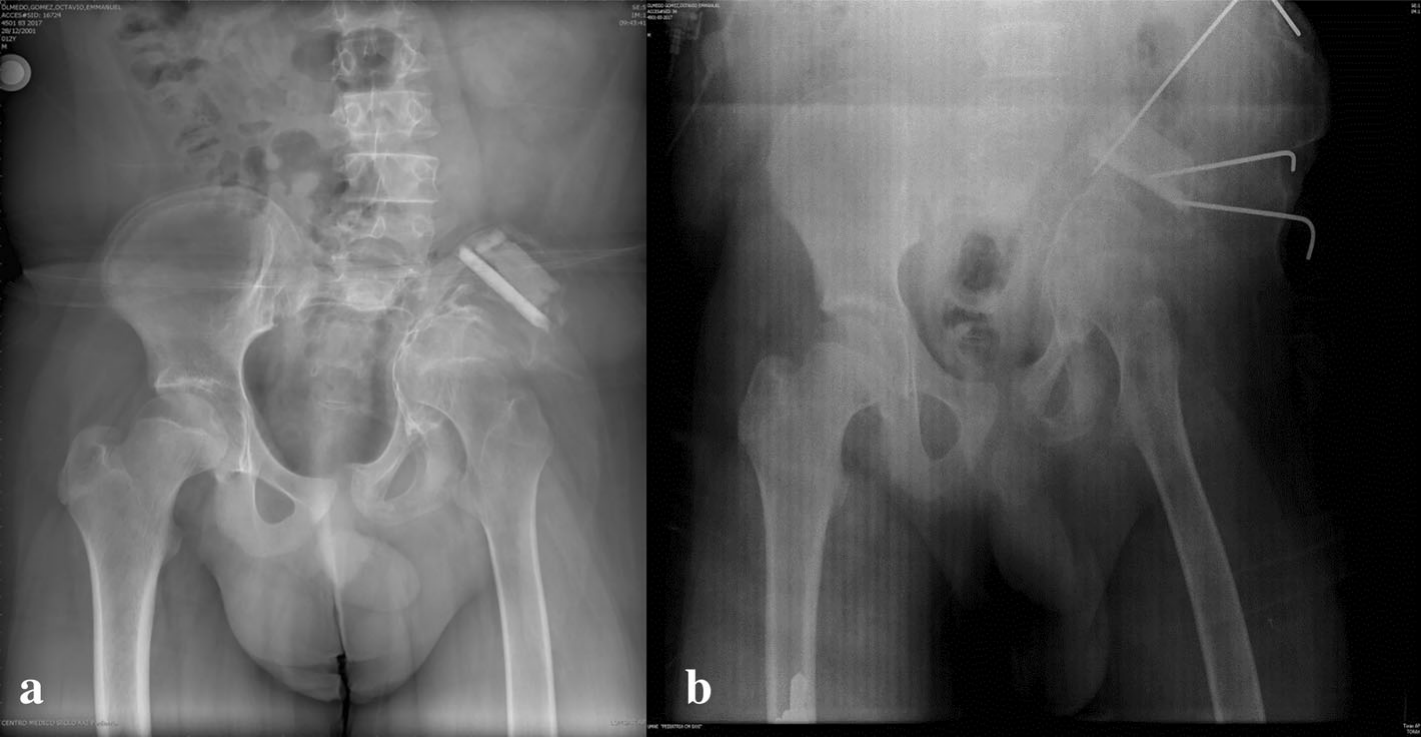
Anteroposterior pelvis X-rays after surgical procedures on the left iliac bone with application of bone graft. **a** November 29th, 2013; **b**) July 1st, 2015, immediate postoperative

**Fig. 3:**
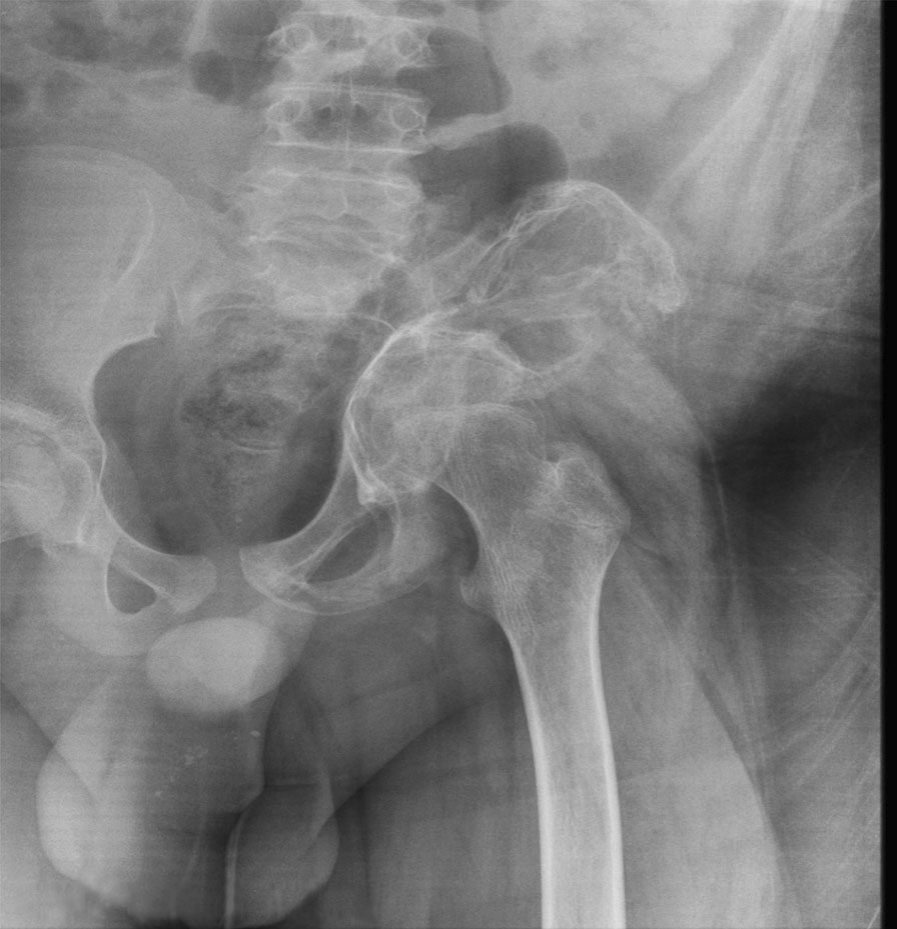
Anteroposterior pelvis X-ray with permeative osteolysis, complete destruction of the left sacroiliac joint and the lower third of the ipsilateral iliac bone, in addition to radiolucency of the femoral neck

Additional studies were performed showing osteolysis of the left iliac bone. Suspecting a malignant process, he was referred to the UMAE at Pediatrics Hospital National Medical Center “Siglo XXI” to the Oncology Department for study protocol at 11 years, 11 months old. Further studies were performed with pelvic radiographs and computerized tomography with three-dimensional reconstruction (see Figs. 1, 2 and 3). The bone scan was negative for infectious or inflammatory bone disease. The magnetic resonance showed a neoplastic lesion of the pelvis with edema, suggestive of Ewing’s sarcoma. An incisional biopsy was performed in December 2013, with an initial histopathology report of an aneurysmal bone cyst. However, the observed osteolysis in the radiographic studies created diagnostic doubts.

Follow-up was performed through the outpatient clinic, showing slight improvement of his symptoms. In June 2014, a new bone biopsy and curettage was performed, with the use of lyophilized cadaveric tricortical bone grafts fixed with Kirschner wire. The histopathological study reported necrosis and reabsorption of spongy bone tissue and vascularized fibrous connective tissue with some osteoclastic giant cells. Two years later, lyophilized cadaveric tricortical bone grafts were used again. He had appropriate evolution and remitting symptoms; thus, he was discharged from the service (Additional file 1).

## Case presentation no. 2

A male patient aged 6 years, 7 months presented with cervical lymphadenopathies in 2011 and was initially treated with antibiotics due to suspicion of an infection, without improvement. Two years later, a cervical lymph node biopsy was performed, reporting a lymph node with diffuse follicular hyperplasia without granules or microorganisms. Neck right lateralization and limited flexion were added. He was assessed by the Neurosurgery Department, who diagnosed the patient with Arnold Chiari type I malformation and performed a suboccipital craniectomy with dural plasty.

**Fig. 4:**
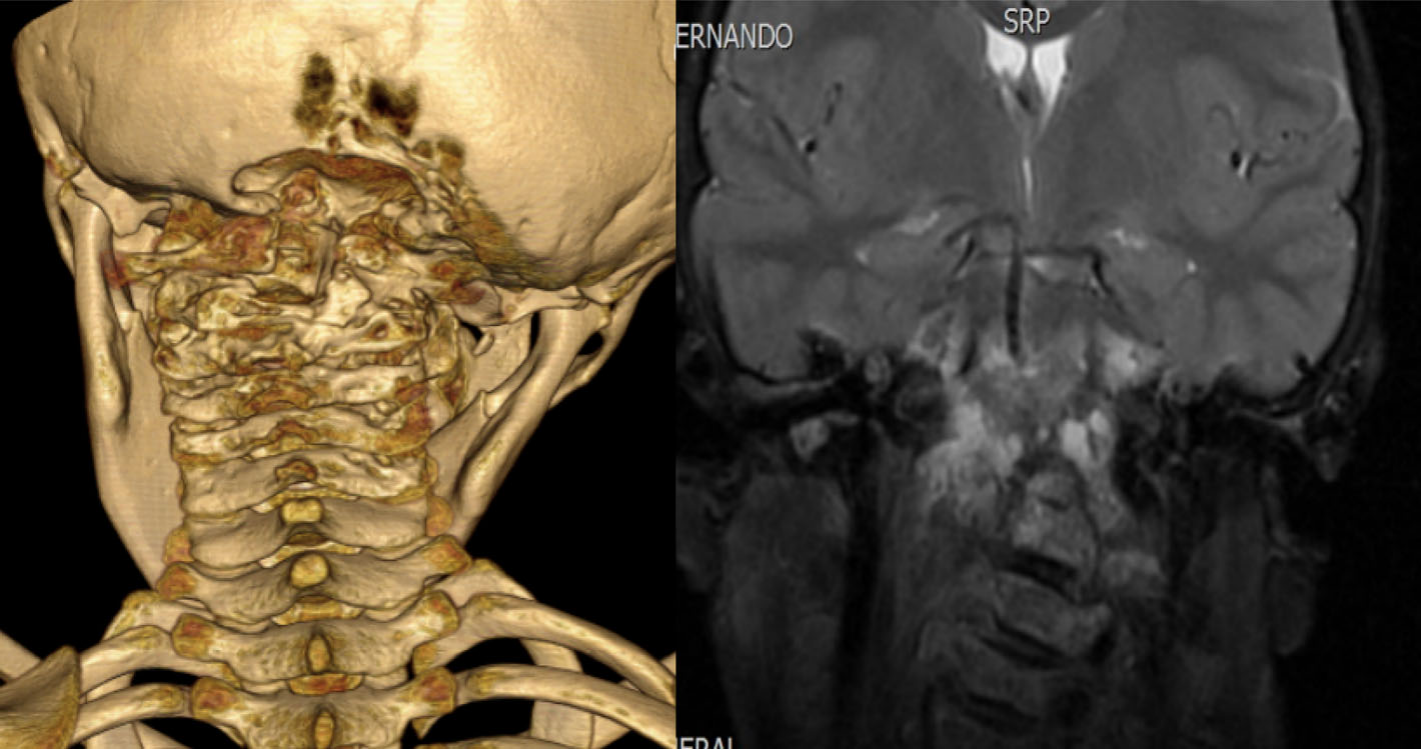
Computed tomography of the cervical spine with three-dimensional reconstruction and bone damage of C1-C3

An additional study protocol was implemented for suspected bone tuberculosis; PCR testing for *Mycobacterium tuberculosis* was performed, with a negative result. Infectious, malignant and systemic etiology were ruled out. A new lymph node biopsy was performed, with a report of nonspecific lymphoid hyperplasia with spots of lymphadenitis that were negative for malignancy. He was hospitalized for 12 days; imaging studies were performed, which showed erosion of the vertebral bodies in the cervical region C1-C3 and skull base (see Fig. 4). An independent slide revision of the second biopsy was requested, reporting bone angiomatosis. In addition, an angiography was performed in June 2016 to assess embolization; however, very small vessels were found.

The patient was evaluated by the Genetic, Endocrinology and Orthopedics Departments for comprehensive management. Treatment with vitamin D and zolendronic acid was started. Additionally, the patient was treated with propranolol. The case was followed up in the Pediatrics Orthopedic Department, and he was hospitalized for a week due to the presence of intense pain in the cervical region that limited walking. Radiotherapy was started in December 2016 with a total dose of 45 GY in 25 fractions, achieving remission of symptoms. He progressed without neurological deficit. Discharge was decided with the use of a Philadelphia-type collar and treatment with bisphosphonates. He was followed up and was kept in the protocol to assess surgical treatment. In 2017, he was admitted to the Emergency Department due to signs of spinal cord compression and died.

## Discussion

Gorham-Stout disease is a rare condition that is difficult to opportunely diagnose and treat. Diagnosis is made by exclusion of other diseases, particularly lymphadenopathies, such as lymphangioma, angiosarcoma, essential or hereditary osteolysis, and bone manifestations of systemic diseases, such as rheumatoid arthritis, syphilis, aseptic necrosis, and hyperthyroidism [8, 18].

As stated in previous studies, the following criteria are required to establish the diagnosis of this disease: 1) radiographic detection of osteolysis; 2) exclusion of cellular atypia; 3) absence of osteoblastic reaction; 4) detection of progressive local injury; 5) exclusion of ulcerative lesions; 6) exclusion of concomitant visceral disease; 7) positive histological tests for proliferation and angiomatous dysplasia; and 8) exclusion of infectious, hereditary, metabolic, neoplastic, and immunological etiology [7, 19]. The two pediatric cases presented here met all these criteria, compatible with nonhereditary type IV osteolysis. However, in a recent study, Ozeki et al. found overlapping features and no histopathological differences between Gorham-Stout disease and other complex lymphatic anomalies; thus, the absence of specific findings in the histopathological study increases the complexity of diagnosing the disease [20].

The clinical manifestations of Gorham-Stout disease located in the spine may include local pain, edema of the affected region, low back pain, pathological fractures, paresthesias, functional alterations and paralysis [21, 22]. The clinical course varies from being self-limiting in some years, with mild manifestations, to a fatal outcome such as the second case study we presented here, where the patient died as a consequence of spinal cord compresion [22].

The exact physiopathogenesis of this entity has not yet been fully elucidated. Several studies have shown that vascular endothelial growth factor (VEGF-A) and interleukin-6 may be elevated in the peripheral circulation of affected individuals, as well as other biomarkers, such as erythrocyte sedimentation rate and alkaline phosphatase [21–23]. Other studies have shown an increase in the concentration of platelet-derived growth factor (PDGF-BB), which plays an important role in the pathogenesis of lymphedema and lymphangiogenesis in tumors [24–34]. There are theories based on cellular and humoral changes as well as the role of angiogenic factors [26].

On the other hand, the surgical options described for this disease include bone resection, resection with endoprosthetic reconstruction and resection with biological reconstruction (bone graft) [10–17]. In patient number 1, biological reconstruction was performed on three occasions, with a remission of symptoms. The patient had an appropriate evolution, and he was later discharged.

Patient number 2 debuted with lymphadenopathies in the cervical region followed by the presence of cervical lytic lesions. To our knowledge, this form of presentation with adenopathies has not been described in the literature. The approach to lymphadenopathies in pediatric patients is highly variable, and the majority are of an infectious and self-limiting etiology [27]. This patient represented a challenge in diagnosis. A cervical biopsy was performed twice, and infectious and malignant etiology was ruled out. Furthermore, the patient was not a candidate for embolization, so treatment with bisphosphonates and radiotherapy was started, with an adequate but partial response. He remained in follow-up, as well as in the protocol for surgical treatment. However, he died due to spinal cord compression. It has been reported that the surgical treatment of patients with this disease presenting spinal involvement is recommended in cases where there is severe pain, pathological fractures, paresthesias, functional alterations and paralysis [28, 29]. Moreover, spinal involvement has been associated with a high mortality (53%) [8], consistent with what happened in the second case presented here. The study by Schuzhong et al. in 2018 reported a case of a 31-year-old male patient with a diagnosis of Gorham-Stout disease, presenting L5 involvement, who was successfully treated with vertebroplasty and application of bone cement. It should be noted that levels of lumbar and sacral involvement have a lower risk of complications, in contrast to osteolysis at higher levels, such as the cervical region, although they are not exempt due to the risk of spinal instability [8, 30].

There are only 5 cases of pediatric patients with Gorham-Stout disease with spinal involvement reported in the literature in the last 10 years, with variable evolution from mild symptoms to death. However, no previous cases with involvement at the cervical level were found [29, 31–34].

One of the situations we would like to highlight with the presentation of these two pediatric reports of Gorham-Stout disease is the fact that, despite their attendance at a tertiary referral pediatric hospital, limited conditions for diagnosing and treating patients were observed. Diagnosis in both cases was delayed because the study protocol was not started when the patients presented with their first symptoms. Furthermore, when they arrived at our hospital, the disease was not suspected from the beginning by the departments where the children initially presented. This evidence suggests that more attention should be given to this rare condition and could offer better treatment options to the patients. Additionally, we consider it very important to continue reporting pediatric cases with this disease to add to the description of affected sites and the severity of the symptoms. Notwithstanding, further clinical and genetic research is mandatory for elucidating the origin and physiopathology of the disease and determining more effective treatments.

## Conclusions

Diagnosis of Gorham-Stout disease is by exclusion; its clinic presentation varies widely, from spontaneous remission to a fatal outcome. Orthopedic surgery continues to be the treatment of choice for pathological fractures of patients with Gorham-Stout disease; however, the results are variable. Bone grafts can be useful, although they do not seem to stop the disease and are also affected by osteolysis.

## Additional file

Additional file 1: He had appropriate evolution and remitting symptoms; thus, he was discharged from the service. (PPT 7872 kb)

## Data Availability

Data generated and analyzed during the current case report are not publicly available due to concerns regarding patient confidentiality. However, the data are available from the corresponding author on a reasonable request.

## Abbreviations

PDGF-BB: Platelet-derived growth factor
UMAE: High Specialty Medical Unit
VEGF-A: Vascular endothelial growth factor
