# Supplementary material for: A report of two children with Gorham-Stout disease
Source: BMC Pediatr. 2019 Jun 24;19:206. doi: 10.1186/s12887-019-1561-0 (PMC6591827; doi:10.1186/s12887-019-1561-0)
Supplement: Supplementary file 1 — He had appropriate evolution and remitting symptoms; thus, he was discharged from the service. (PPT 7872 kb) [file 12887_2019_1561_MOESM1_ESM.ppt]

## Slide 1
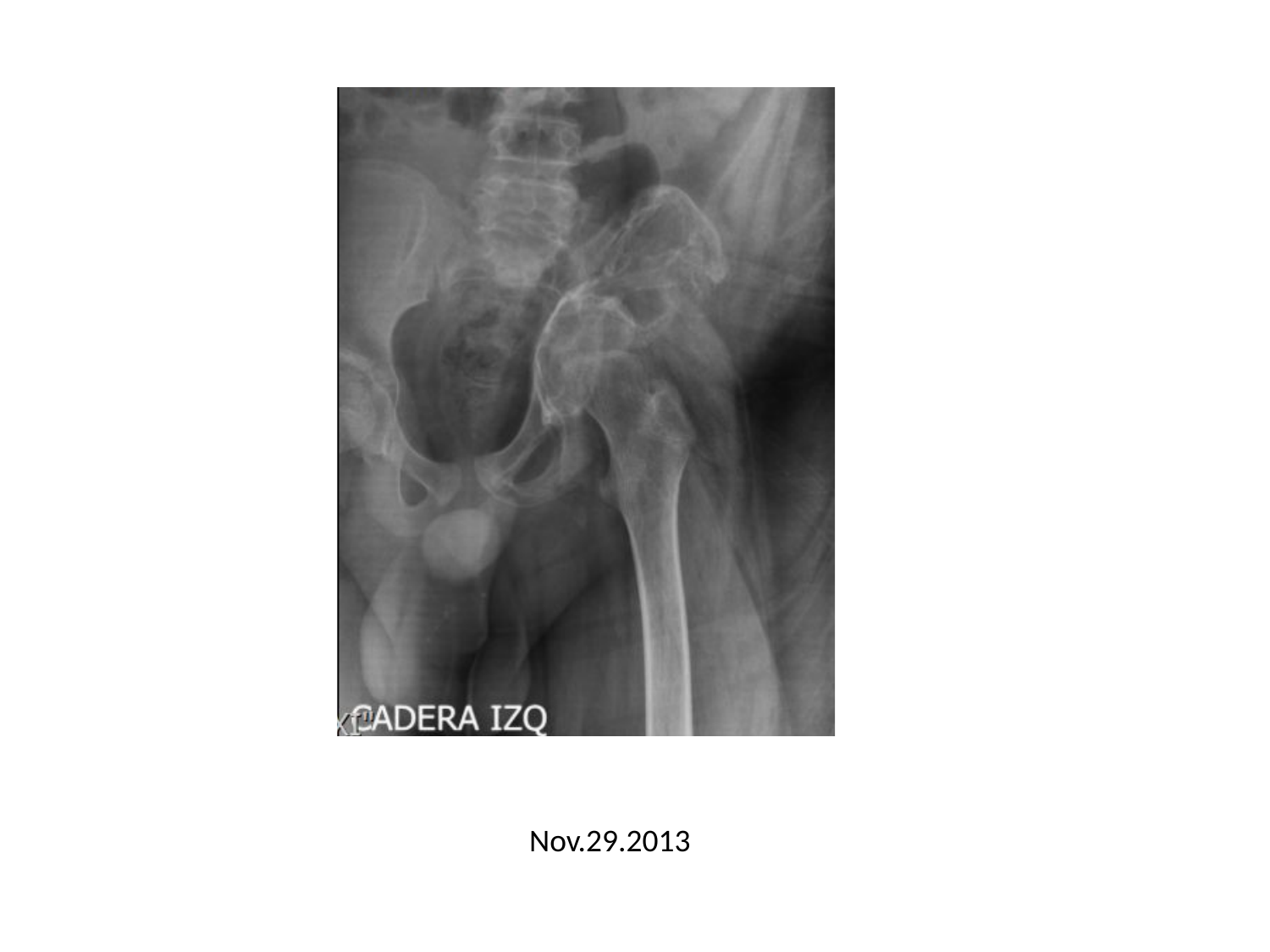

Nov.29.2013

## Slide 2
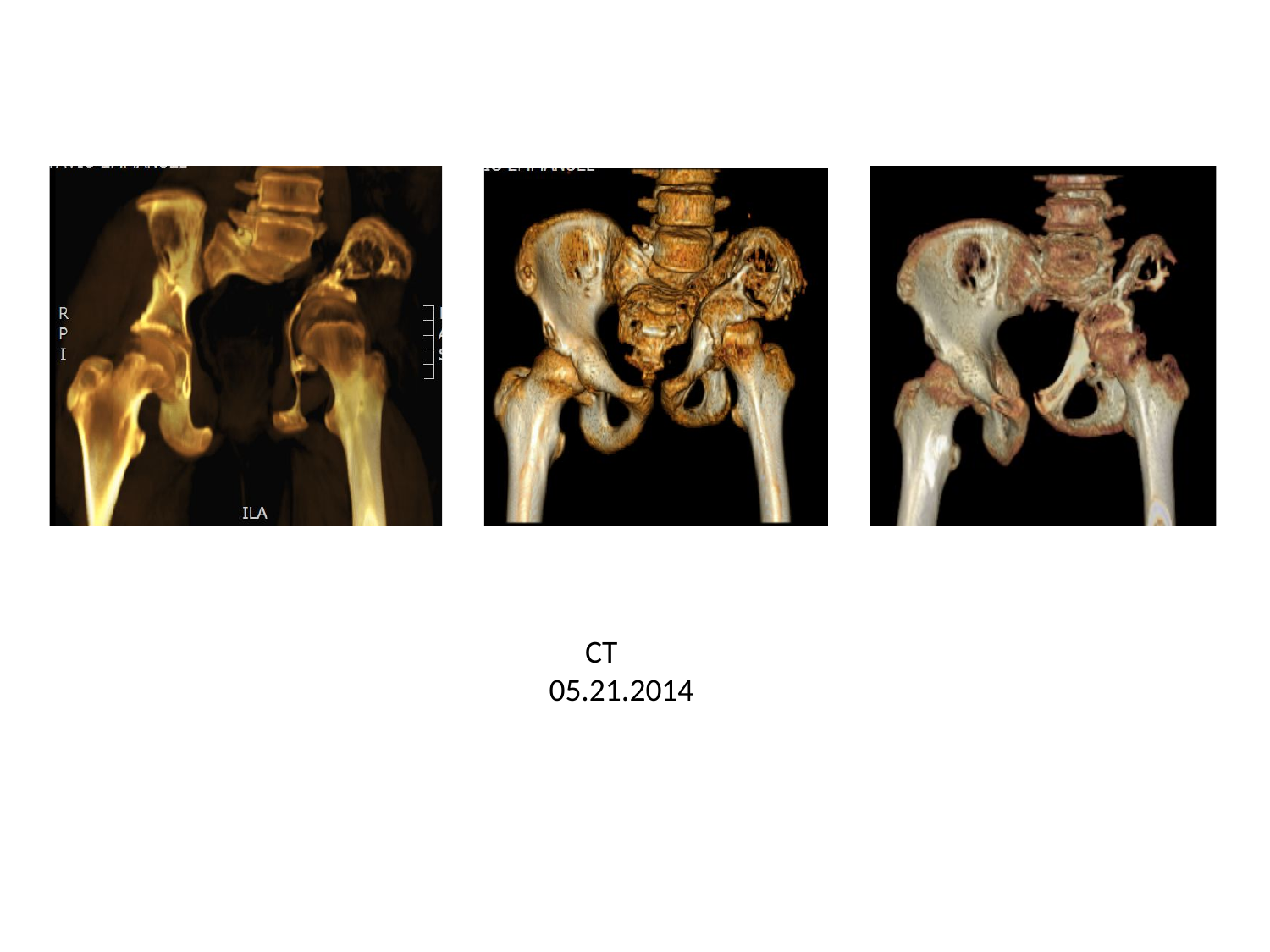

CT
05.21.2014

## Slide 3
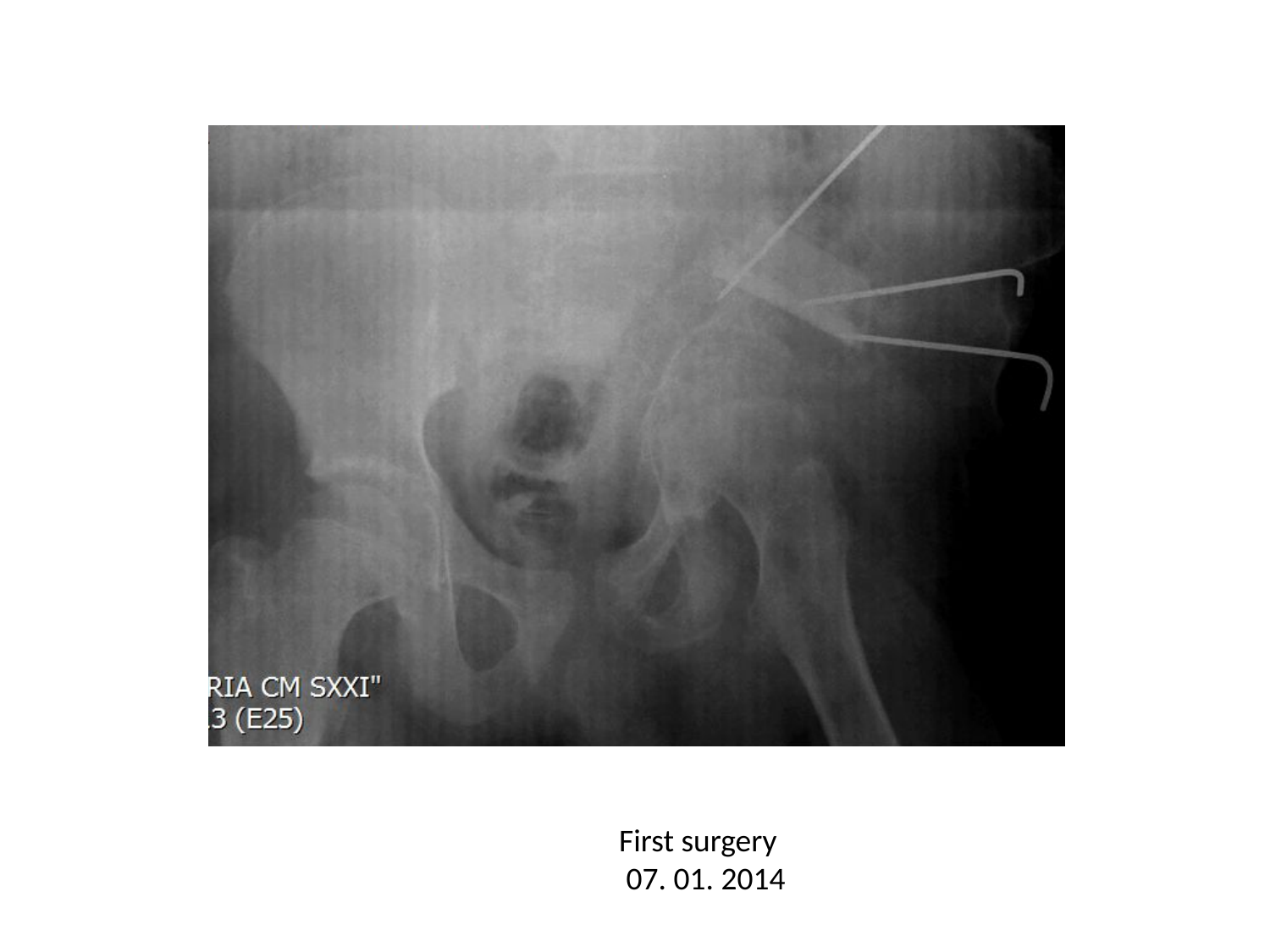

#
First surgery
 07. 01. 2014

## Slide 4
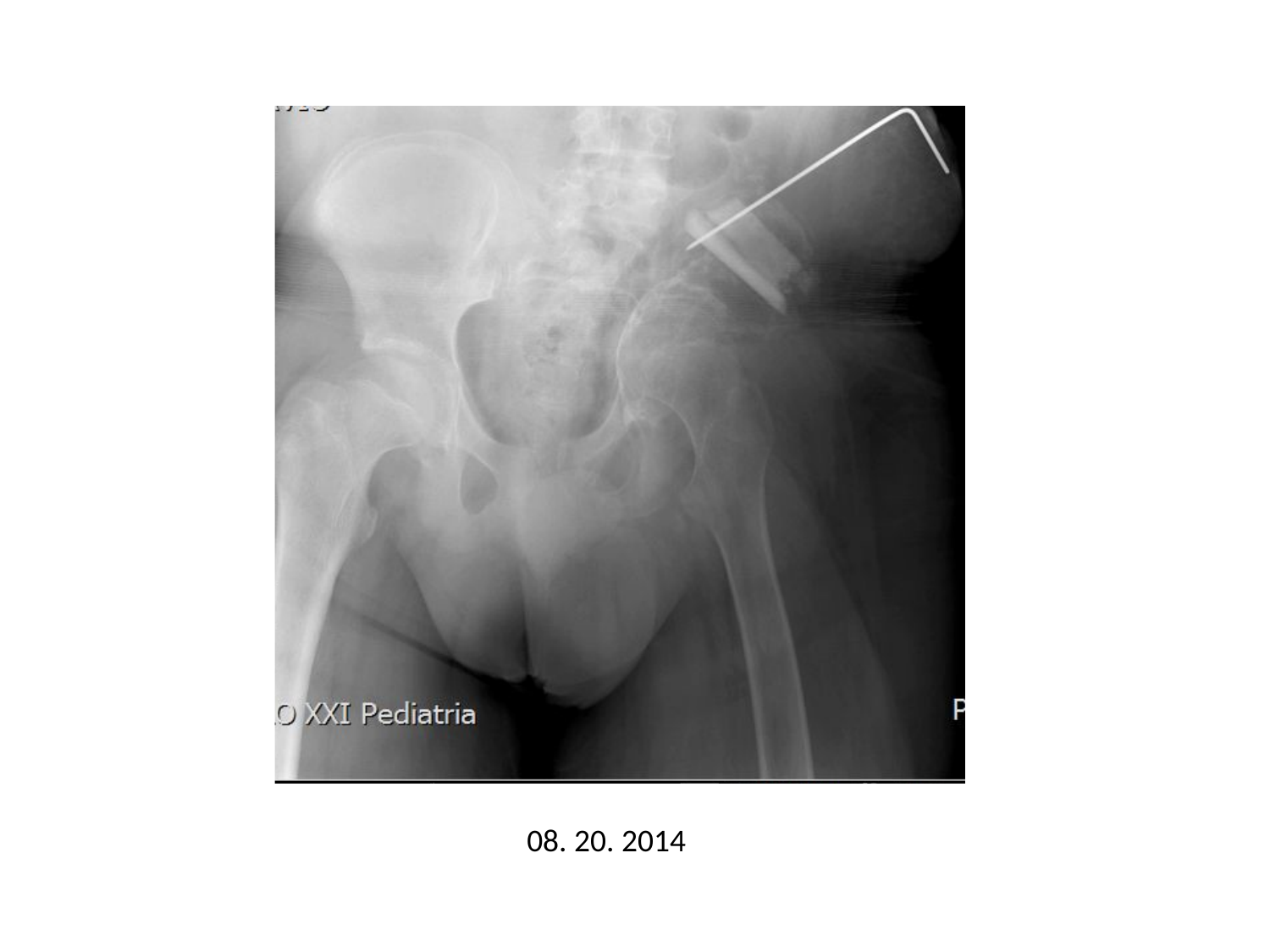

08. 20. 2014

## Slide 5
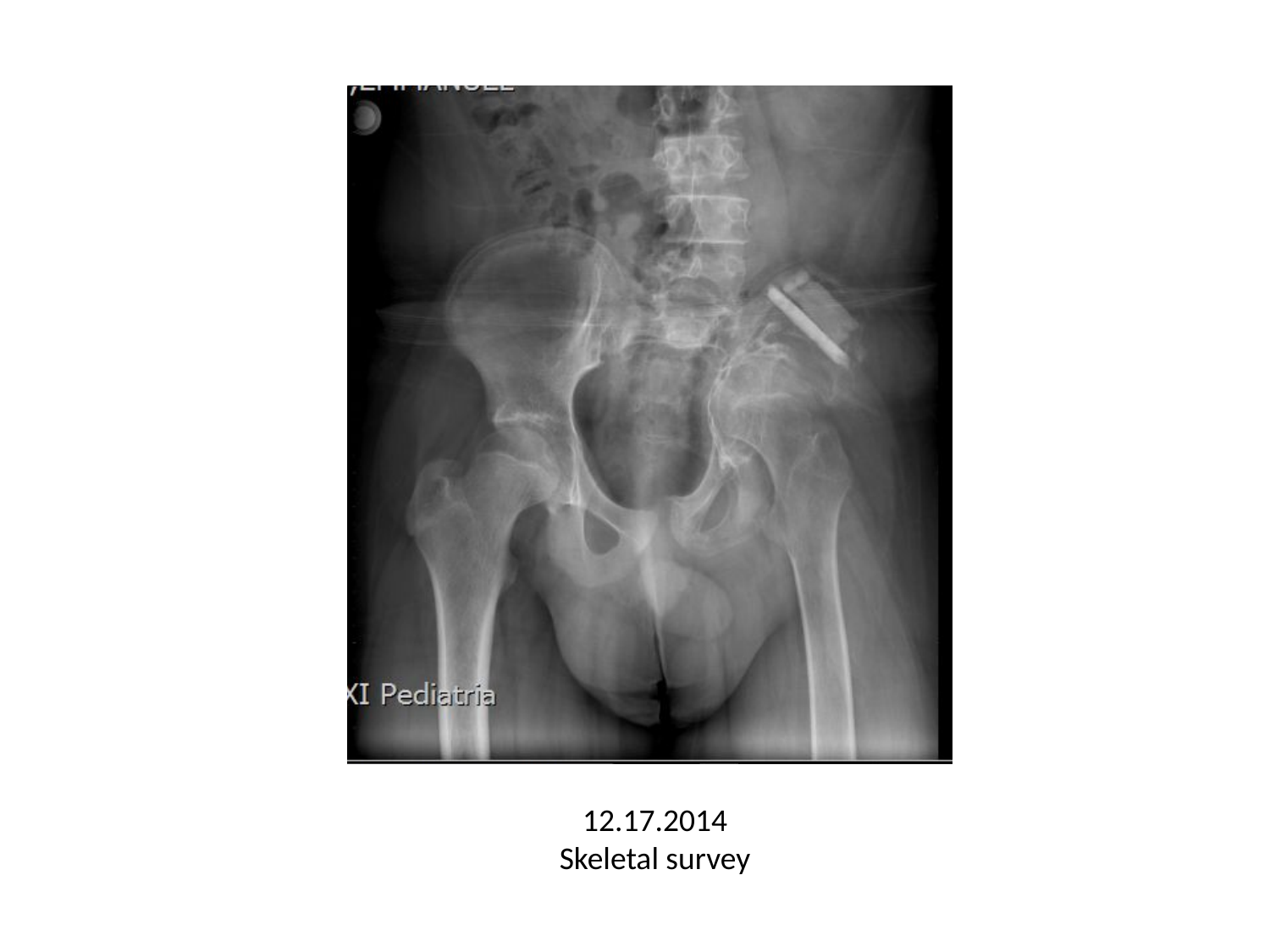

12.17.2014
Skeletal survey

## Slide 6
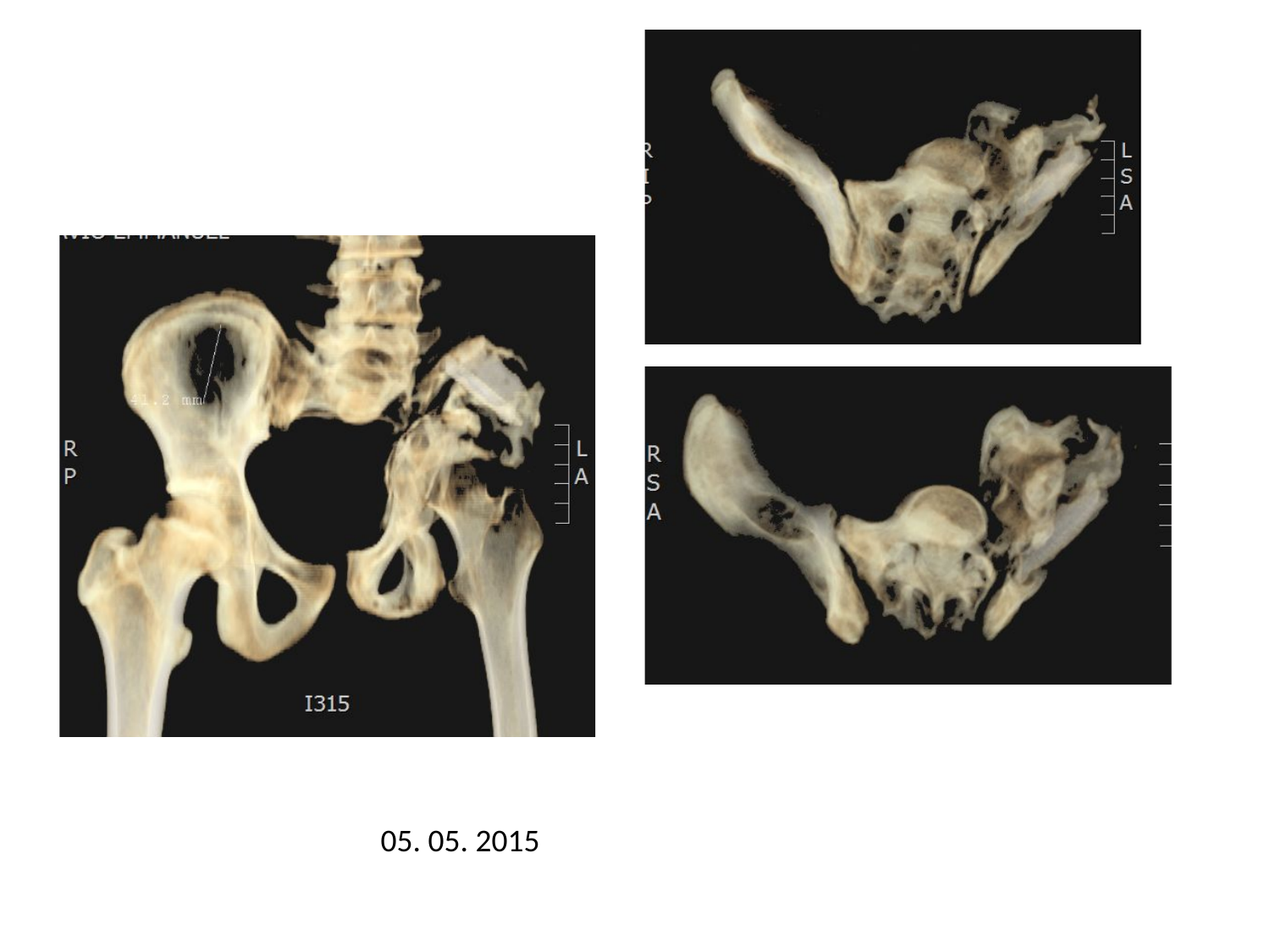

05. 05. 2015

## Slide 7
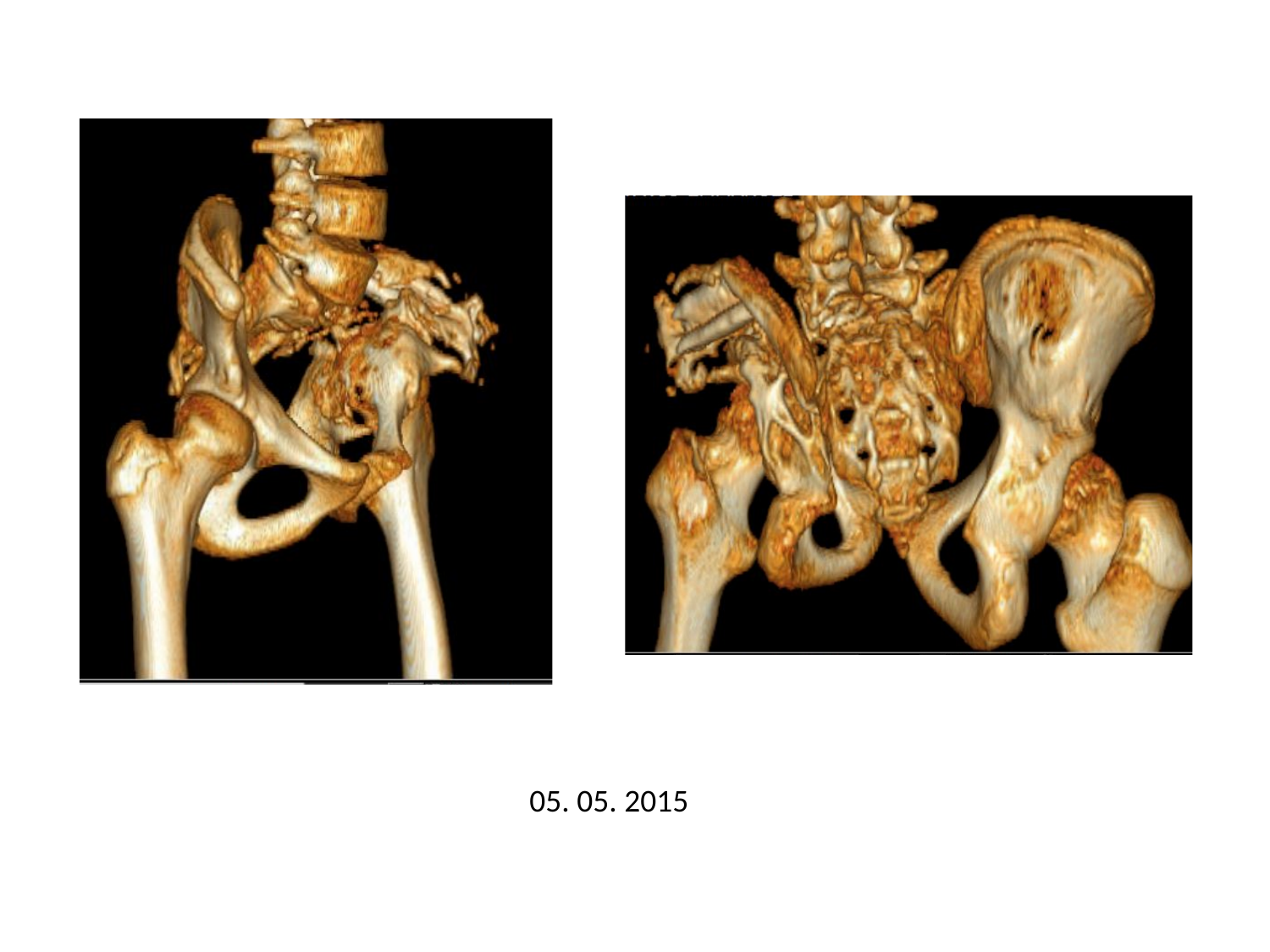

05. 05. 2015

## Slide 8
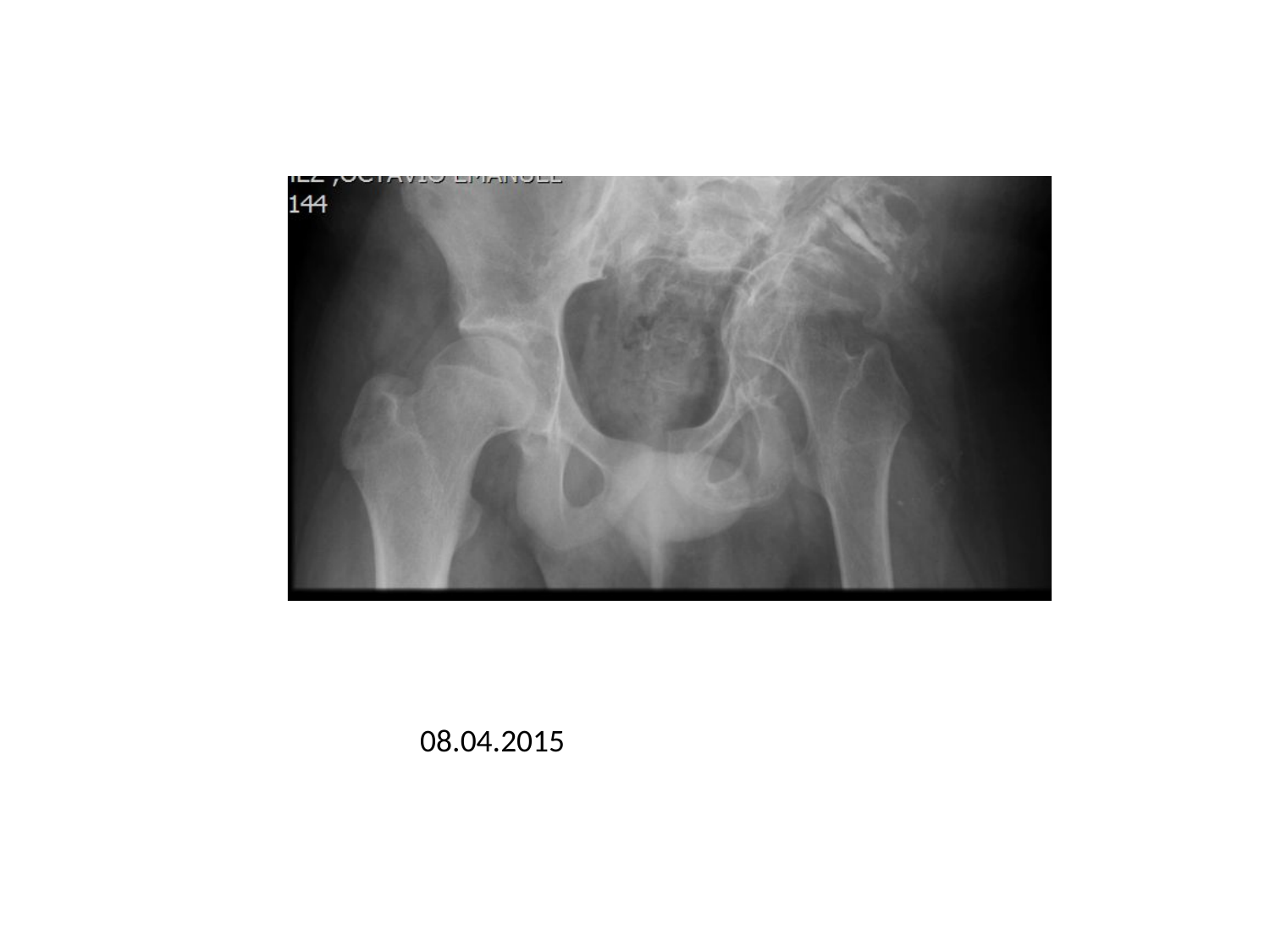

08.04.2015

## Slide 9
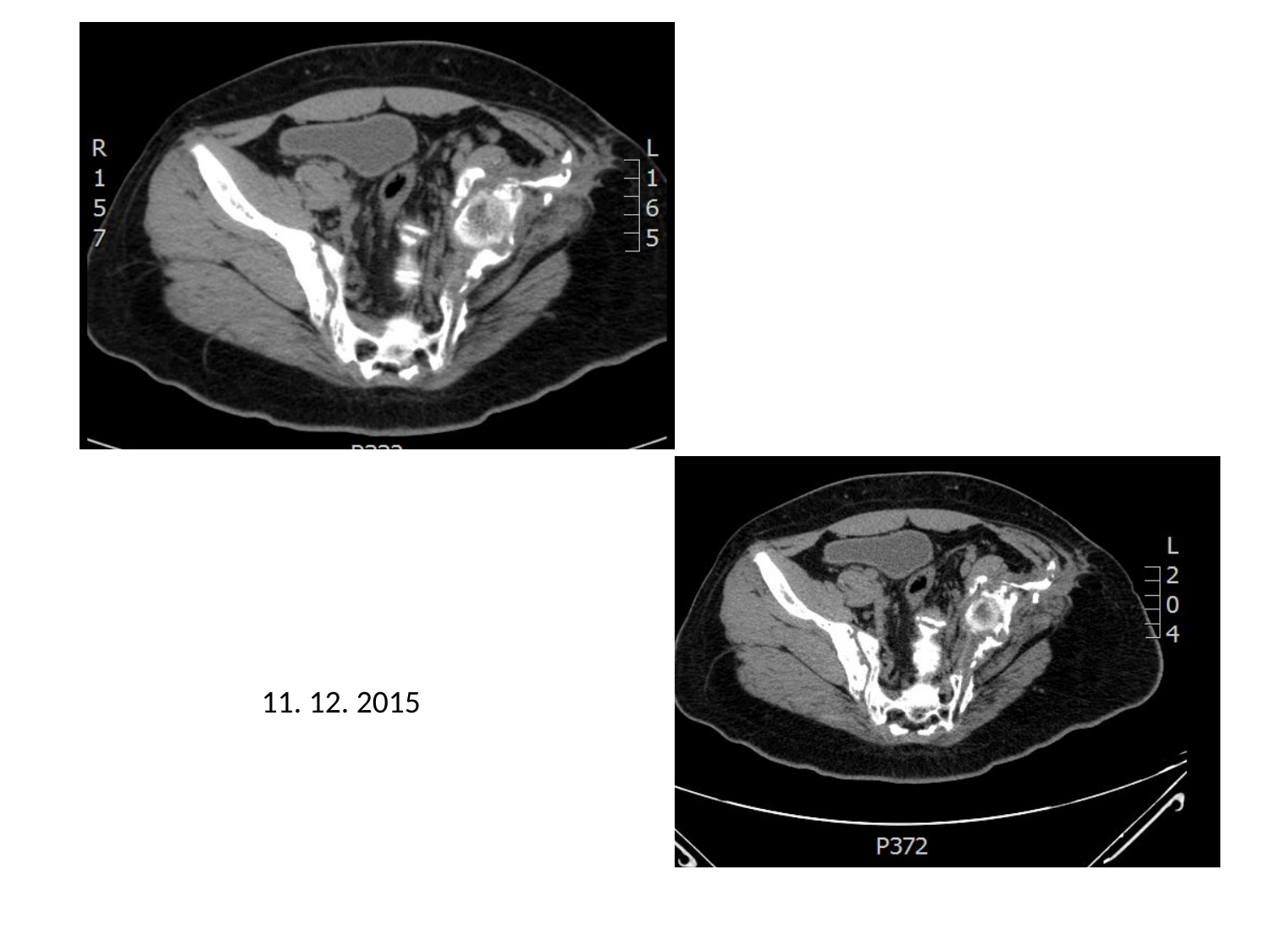

11. 12. 2015

## Slide 10
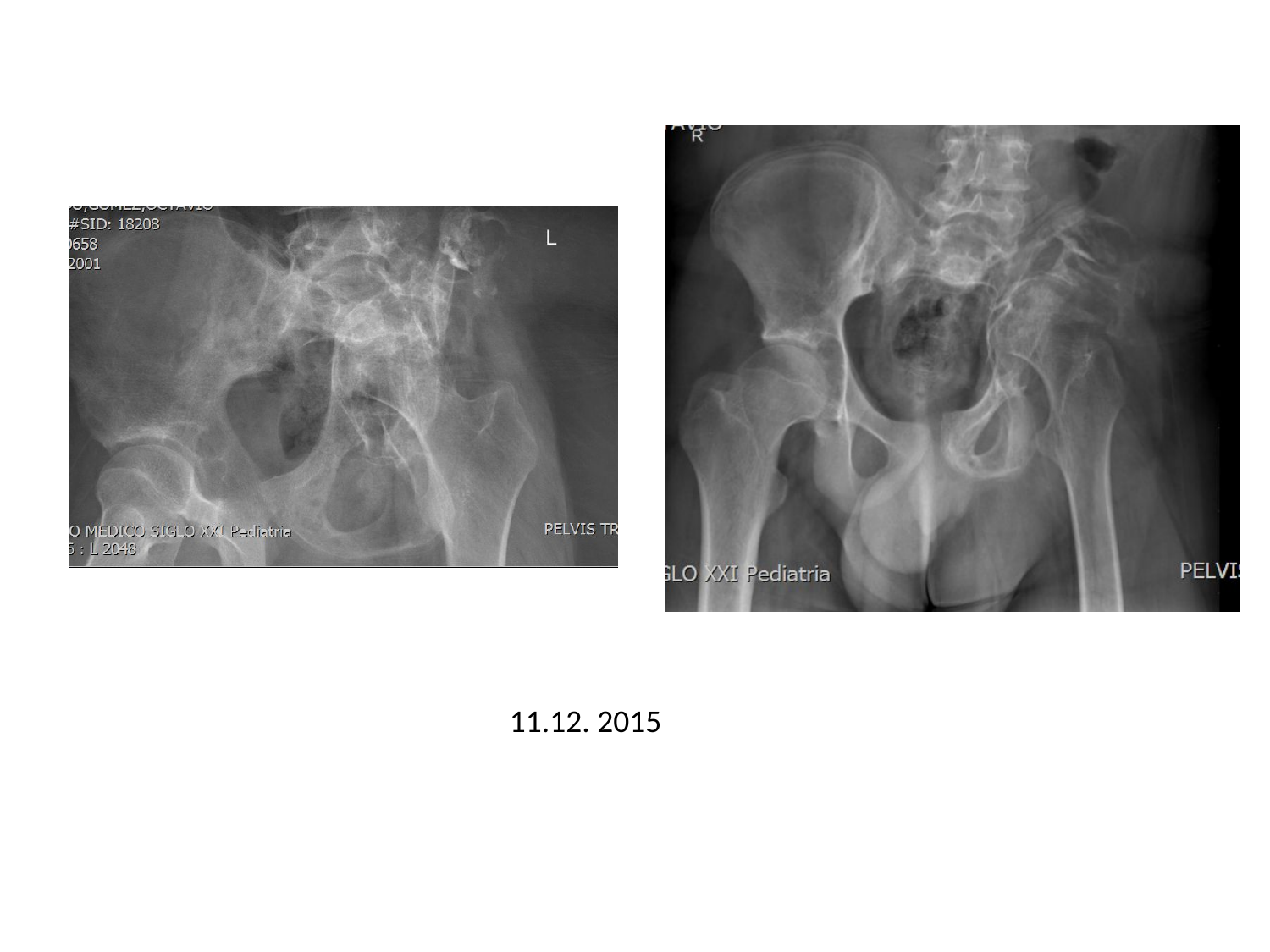

11.12. 2015

## Slide 11
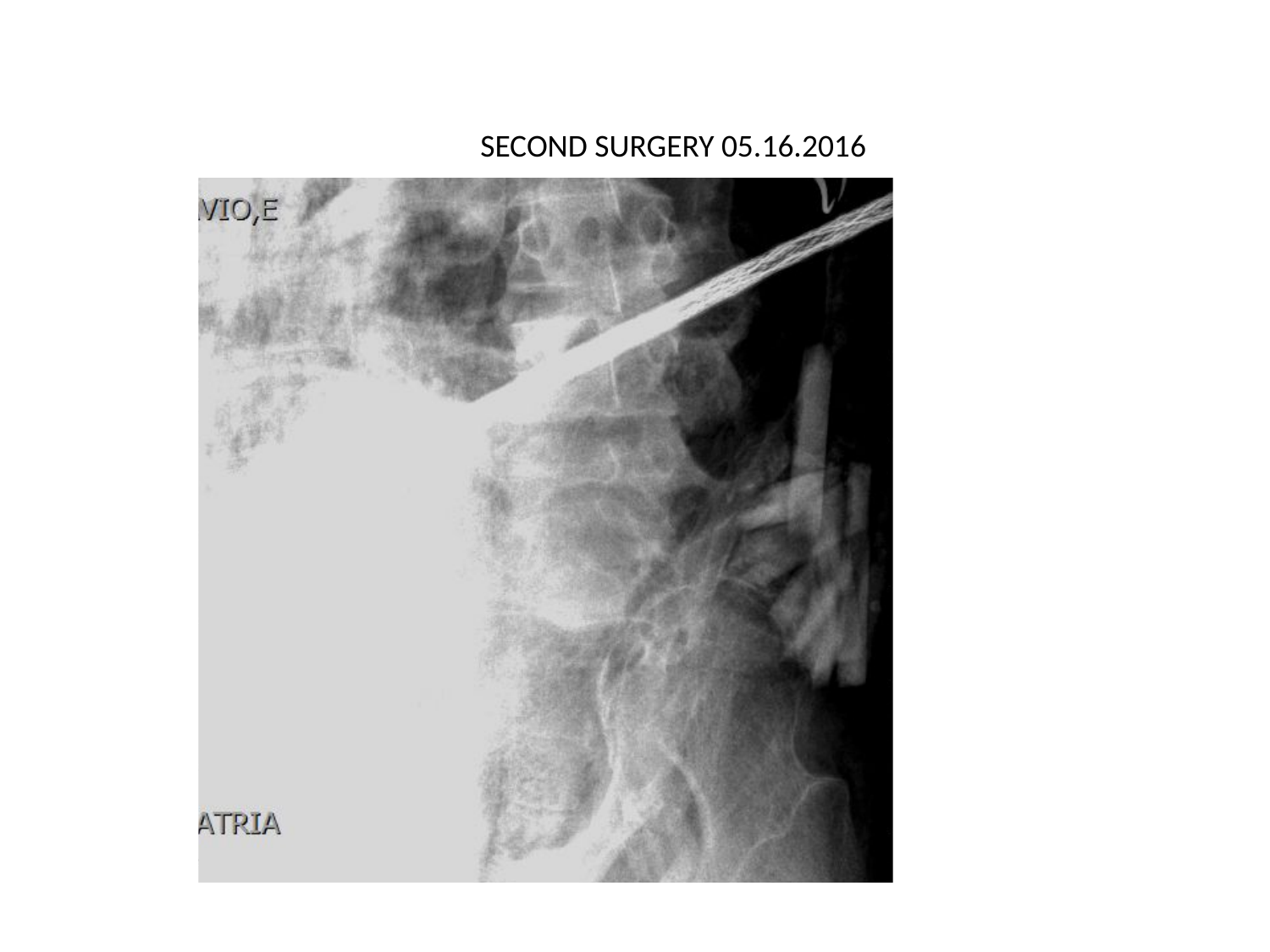

SECOND SURGERY 05.16.2016

## Slide 12
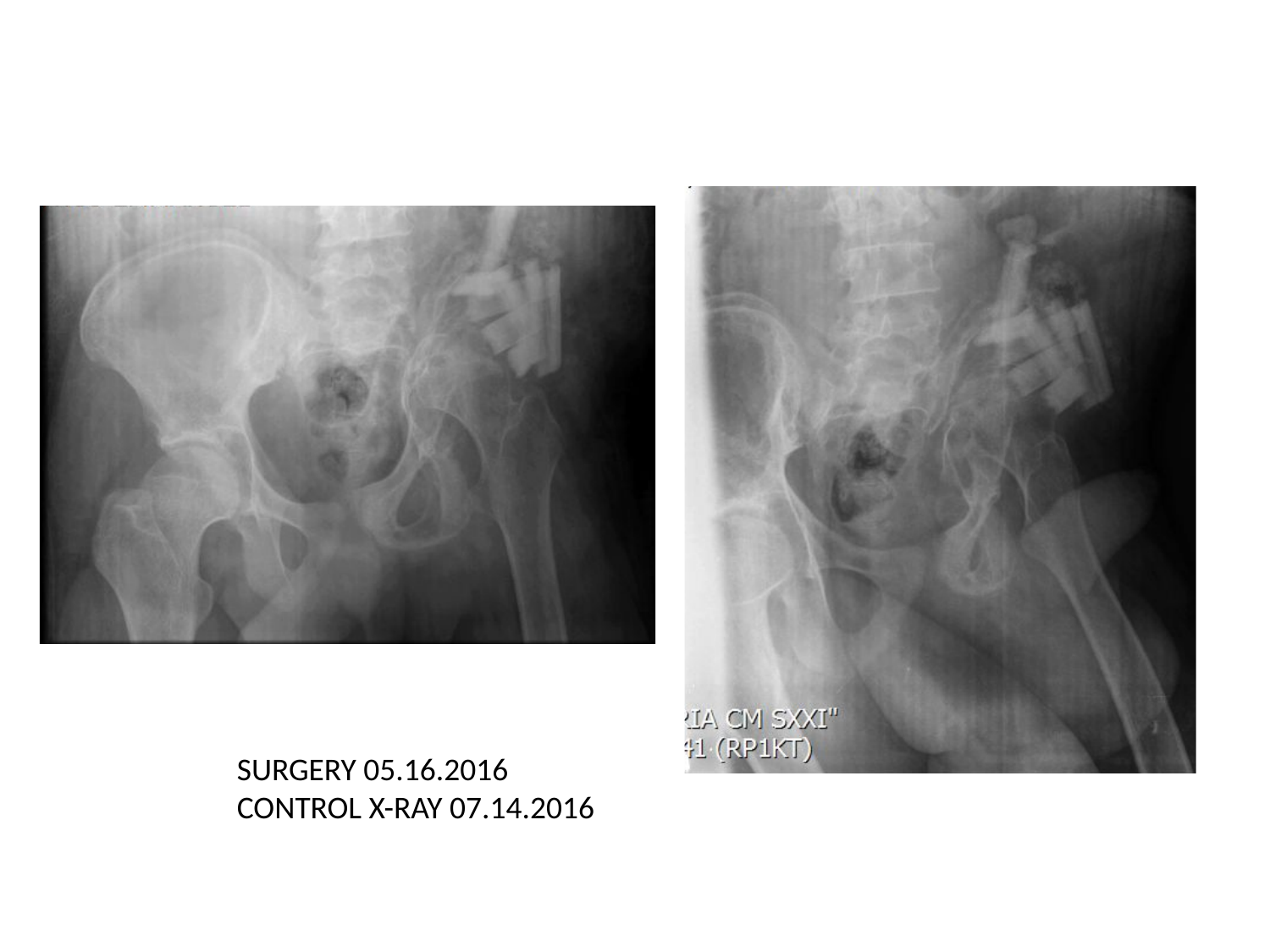

SURGERY 05.16.2016
CONTROL X-RAY 07.14.2016

## Slide 13
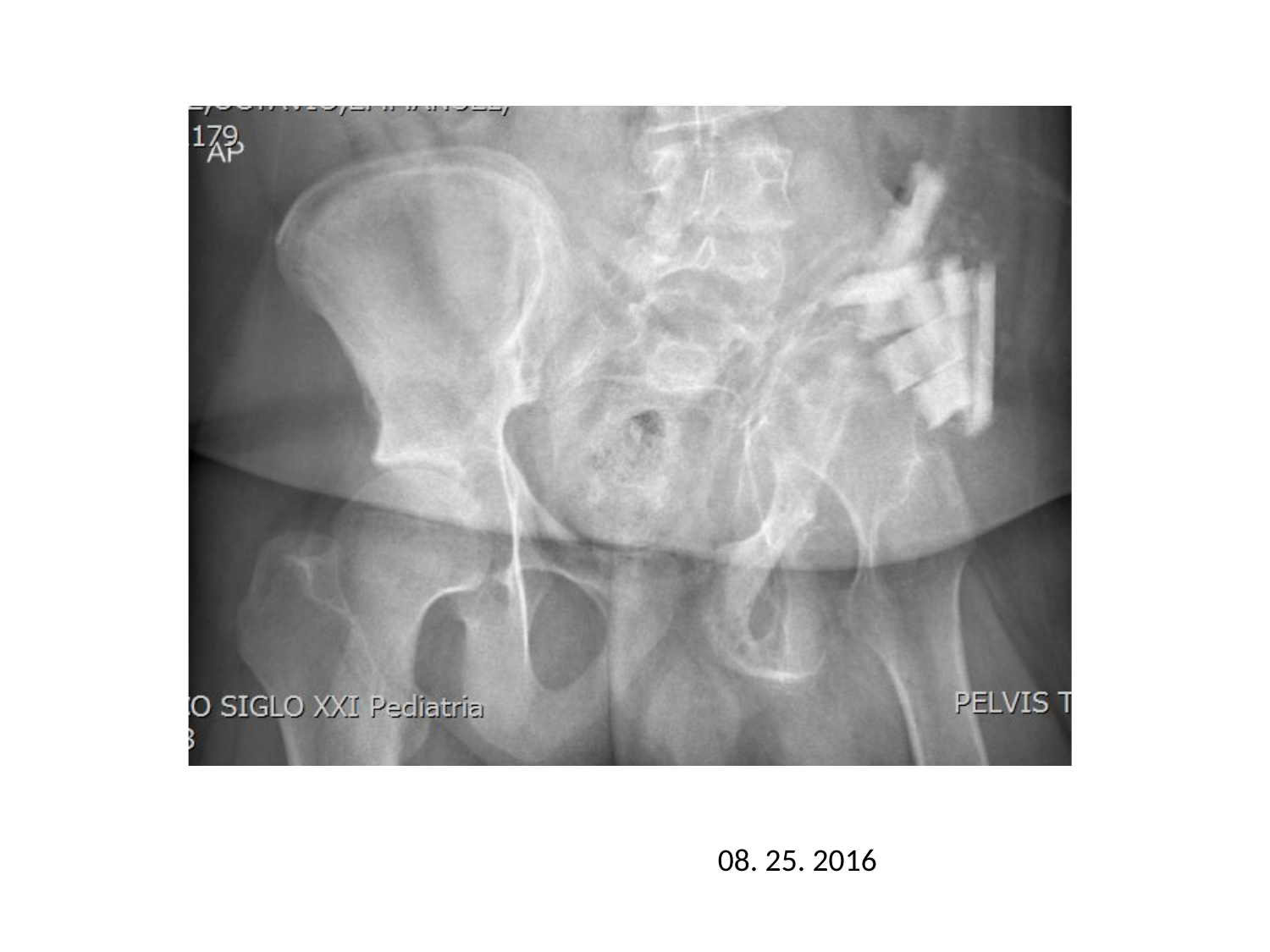

08. 25. 2016

## Slide 14
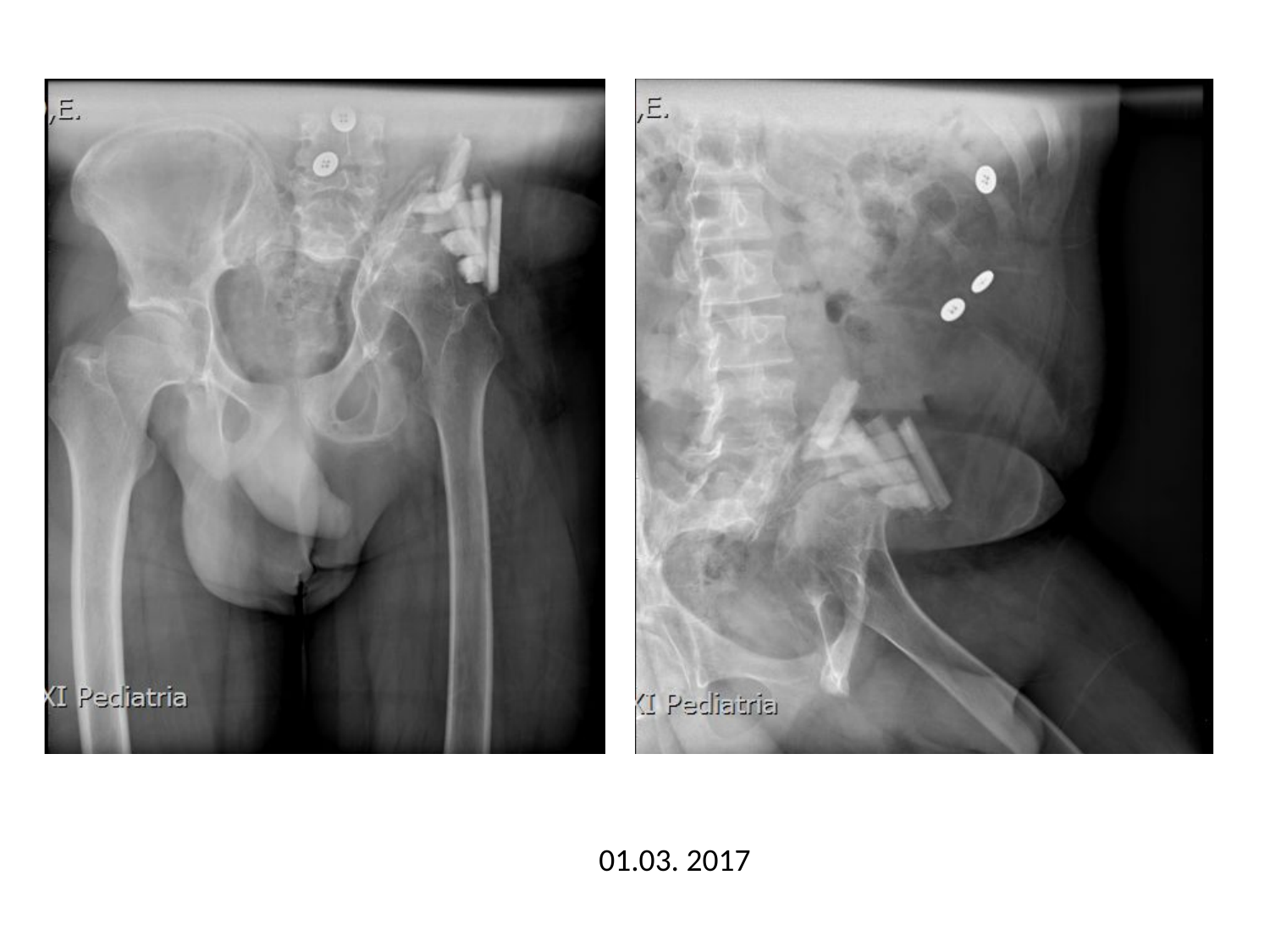

01.03. 2017

## Slide 15
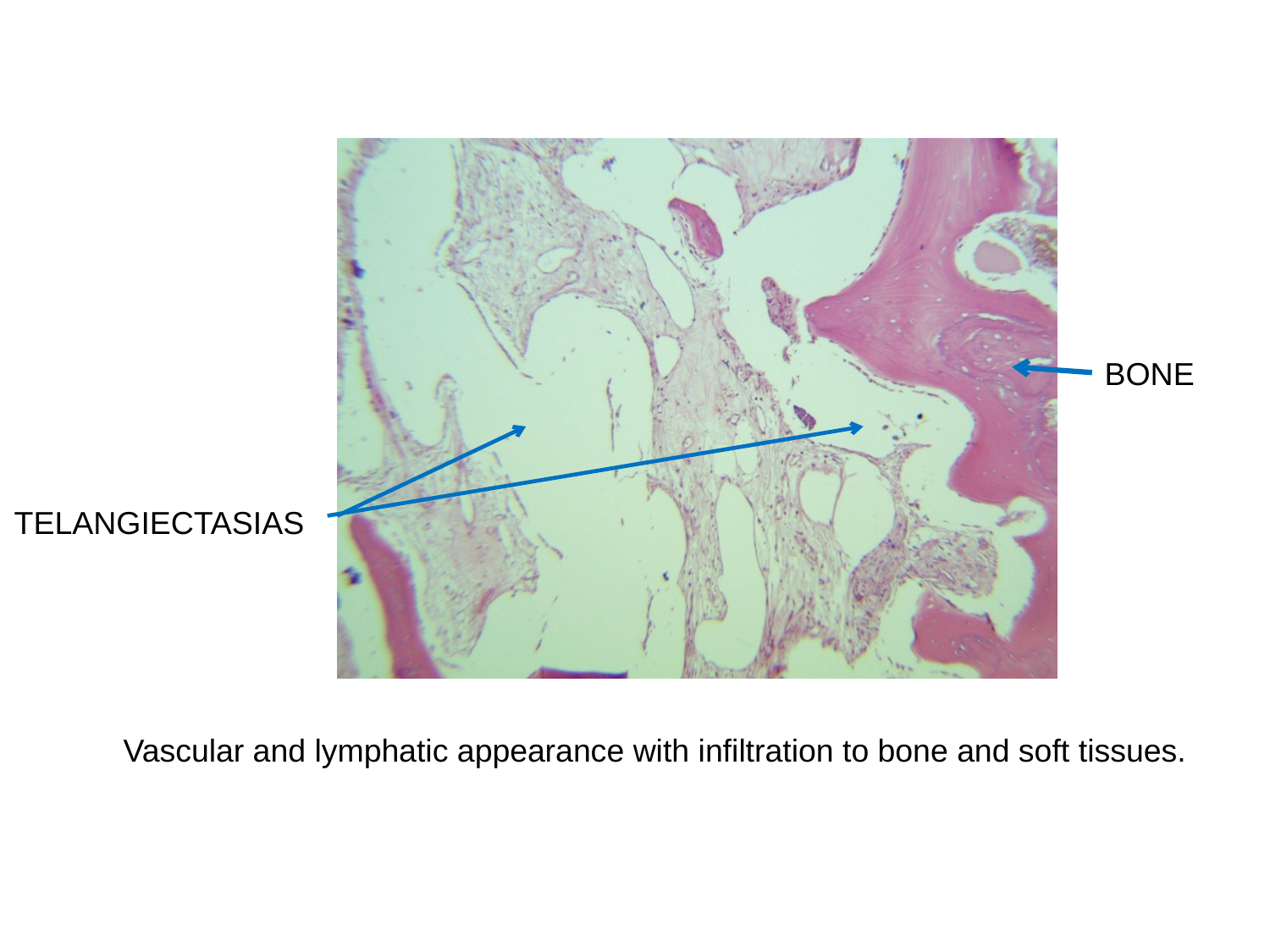

BONE
TELANGIECTASIAS
Vascular and lymphatic appearance with infiltration to bone and soft tissues.
